# The individuation of mathematical objects

**DOI:** 10.1007/s11229-024-04814-6

**Published:** 2024-12-23

**Authors:** Bahram Assadian, Robert Fraser

**Affiliations:** 1 School of Philosophy, Religion and History of Science, University of Leeds, Woodhouse Lane, Leeds, LS2 9JT UK; 2 Department of Politics, Philosophy and International Relations, Swansea University, Swansea, UK

**Keywords:** Identity, Individuation, Grounding, Mathematical objects, Mathematical platonism

## Abstract

Against mathematical platonism, it is sometimes objected that mathematical objects are mysterious. One possible elaboration of this objection is that the individuation of mathematical objects cannot be adequately explained. This suggests that facts about the numerical identity and distinctness of mathematical objects require an explanation, but that their supposed nature precludes us from providing one. In this paper, we evaluate this nominalist objection by exploring three ways in which mathematical objects may be individuated: by the intrinsic properties they possess, by the relations they stand in, and by their underlying ‘substance’. We argue that only the third mode of individuation raises metaphysical problems that could substantiate the claim that mathematical objects are somehow mysterious. Since the platonist is under no obligation to accept this thesis over the alternatives, we conclude that, at least as far as individuation is concerned, the nominalist objection has no bite.

## Introduction

Mathematical platonism is the view that abstract mathematical objects, such as numbers and sets, exist independently of our language, thought, and practices. Mathematical nominalism denies this. Nominalism tends to be motivated by the claim that platonic mathematical objects are, in some way or another, mysterious. One elaboration of this claim is that, given the existence of platonic mathematical objects, facts about their numerical identity and distinctness (facts such as 1=1 and 1 ≠ 0) cry out for an explanation, but the nature of mathematical objects precludes us from providing one. This objection is prior to the more familiar *epistemological* objections that ask how, if at all, mathematical knowledge is possible, given that it is knowledge of abstract objects that cannot enter into any causal relations with us. The objection asks instead how facts about the identity of such objects can possibly obtain in the first place. It, thus, is a *metaphysical* objection.^1^

In this paper, we evaluate this objection. To individuate objects is to ground or metaphysically explain facts about their numerical identity and distinctness.^2^ Thus, the challenge is to find an acceptable way to individuate abstract mathematical objects. To evaluate the objection, we explore three means of individuation: individuation by intrinsic properties (intrinsic individuation), individuation by relations (relational individuation), and individuation by underlying substance (substantial individuation). We argue that only the latter means raises metaphysical problems that might serve to substantiate the nominalist objection, and that the platonist is not obliged to accept this proposal over the others.^3^

We begin by showing that no distinctive metaphysical difficulties arise if mathematical objects are intrinsically and relationally individuated (Sects. 2 and 3). We then show that distinctive metaphysical difficulties *do* arise on the view that mathematical objects are substantially individuated, but argue that this is only a problem if there is pressure for the platonist to accept this view (Sect. 4). We then consider a recent argument for mathematical nominalism from Builes (2022) which threatens to provide such pressure, but we show that the argument fails (Sect. 5). We conclude that, elaborated in terms of individuation, the nominalist objection has no bite (Sect. 6).

Before moving on, some words about the scope and assumptions of this paper. First, we presume a notion of *fundamentality* spelled out in terms of the relation of *grounding*, which connects less fundamental facts with more fundamental facts, such that when one fact (partially or wholly) grounds another, the former is more fundamental than the latter. On this picture, a fact is fundamental if and only if it is ungrounded, and thus, has no metaphysical explanation. We do not assume that grounding is unitary—for all we say, ‘grounding’ may serve as an umbrella term for a range of specific metaphysical dependence relations.^4^ We also make no further commitments regarding the precise connection between grounding and explanation, other than that (partial or whole) grounding is sufficient for metaphysical explanation.

Given this notion of fundamentality, we can provide further elucidation of the objection we are evaluating in this paper. The objection states that identity and distinctness facts regarding platonic mathematical objects require an explanation that cannot be given. There are two important presuppositions at work here. First, it is presupposed that identity and distinctness facts (concerning mathematical objects) are not fundamental, and thus require some metaphysical explanation. Second, it is presupposed that the *nature* or *constitution* of (mathematical) objects is somehow relevant to the explanation of their identity and distinctness.

Both presuppositions can, of course, be questioned. Regarding the first, one might claim that all identity and distinctness facts are fundamental, and thus settled independently of the nature of the different kinds of objects they concern. One might support this claim by appealing to an alternative conception of fundamentality defended by Sider (2011), who argues that fundamental facts are those represented by *joint-carving terms*—terms which represent the objective structure of the world by marking genuine similarities and differences in nature. (Sider draws on Lewis’ (1983) conception of naturalness here.) Importantly, Sider includes first-order quantifiers and the identity predicate among the join-carving vocabulary, meaning that truths about identity and distinctness represent fundamental facts.

However, without further argument, this move does not bear on our presupposition. One can concede that identity and distinctness facts are fundamental in Sider’s sense, representing objective similarities and differences in nature, without accepting that they are ungrounded. Whatever positive reasons there are for thinking that the identity predicate is joint-carving are not reasons for denying that identity and distinctness facts are grounded. Of course, one might suggest that Sider’s notion of fundamentality is *the* notion of fundamentality, and thus discredit the ground-theoretic notion. But the scope of this paper does not extend to providing a general defence of the ground-theoretic conception of fundamentality, so we will not engage with this suggestion here.

The second presupposition was that the nature or constitution of platonic mathematical objects is relevant to their individuation. This too can be questioned. One might take facts about the identity and distinctness of objects to be one thing, and facts about the nature of those same objects to be entirely another. For example, a recent proposal is that identity and distinctness facts are all ‘zero-grounded’, i.e. not fundamental, but not grounded in anything, either.^5^ This is not the place for an in-depth evaluation of this proposal, but it is worth noting that it has counterintuitive consequences. In particular, it takes the fact that *a* = *a* and the fact that *a* ≠ *a* to have the very same ground. As Shumener (2020, p. 422) notes, this does not seem to be a good basis for a satisfying *explanation* of such facts. This provides some pull towards the idea that individuation is tied up with the constitution or nature of objects: the explanation of facts about the identity and distinctness of objects appeals to features of the objects in question.^6^

While they are not uncontroversial, we have seen that there are positive reasons to accept the two presuppositions underpinning the nominalist objection. We are thus content to adopt them as our working assumptions, and proceed to evaluating proposals for individuating platonic mathematical objects in terms of their nature or constitution.

## Intrinsic individuation

Before considering the view that mathematical objects are intrinsically individuated, we must first get clear on what intrinsic properties are. They are supposed to be the ways things are in themselves, rather than in relation to other things. On one popular analysis, a property is intrinsic if and only if it is shared among its exact duplicates. For example, the exact duplicates of *x*’s head will have the same mass as *x*’s head, so its mass is an intrinsic property of *x*’s head. However, in a possible world in which a counterpart of *x* is decapitated, an exact duplicate of *x*’s head is no longer attached to an exact duplicate of *x*’s body. Thus, *being attached to x’s body* is an *extrinsic* property of *x*’s head.^7^

While this analysis captures the notion well enough for ordinary concrete objects, it is not suitable for mathematical objects. For if a mathematical object has no duplicates except itself, then all of its properties will count as intrinsic (Linnebo, 2008, p. 65). But that cannot be right. Mathematical objects do have extrinsic properties. Take the property *being composite*, for example. The fact that 6 is composite is not a matter of how 6 is in itself, but of how 6 relates to other numbers. The same goes for other mathematical properties such as *being even* and *being prime*. Intuitively, then, at least some mathematical properties are extrinsic.

A more promising analysis is that a property is intrinsic just in case an object’s having that property does not *depend on* what other objects exist or how it is related to them. This analysis does not rely on similarities and differences between possible duplicates. For example, even though 6 is composite in every possible world, its *being composite* depends on other things—namely 2 and 3—and so, *being composite* is not intrinsic. Intuitively, and according to this alternative analysis, there are at least some extrinsic mathematical properties. But are there any intrinsic mathematical properties? In what follows, we will suggest that there are no intrinsic mathematical properties of individual mathematical objects. However, we argue that this does not preclude the possibility that mathematical objects have *non-mathematical* intrinsic properties that might serve to individuate them.

According to an increasingly popular view, mathematics studies the *mathematical* and *structural* properties exhibited by various systems of objects, and not the intrinsic properties of the objects that may stand in those relations.^8^ If this view is right, mathematical and structural properties must be relational, and thus extrinsic. (One important caveat: the mathematical properties of a mathematical structure, taken as a whole, are a matter of how its internal ‘parts’ relate to each other, and so, while relational, are arguably intrinsic. For example, a group’s being cyclic is a matter of its being generated by just one of its elements. To give a non-mathematical analogy: *x’s having longer arms than legs* seems to be an intrinsic property of *x*, though it is relational. We accept this. Our claim is that mathematical properties of *individual* mathematical objects are extrinsic, which is compatible with saying that there are intrinsic non- distributive properties of collections of mathematical objects.)

So, we have reason to think that all *mathematical* properties of mathematical objects are extrinsic. However, one should not thereby conclude that mathematical objects lack intrinsic properties. After all, mathematical objects may instantiate *non-mathematical* intrinsic properties. Builes has recently objected to this suggestion:

If the number 2 does have some intrinsic quality, it follows that it must have an intrinsic quality that isn’t investigated by any field of inquiry *at all*, mathematical or otherwise. Such a position seems to be committed to a kind of *mysterianism* about the number 2. If no field of inquiry can investigate the intrinsic qualities of the number 2, then such intrinsic qualities seem unknowable. Not only do we not *know* which intrinsic qualities the number 2 has, but we don’t even have a positive conception of what such an intrinsic quality could possibly be like. (Builes, 2022, pp. 73–4)

This seems unduly pessimistic to us. While we grant that mathematical inquiry does not study intrinsic properties of mathematical objects, we see no reason to assume that *no field of inquiry* is apt for studying them. For example, why not think that metaphysics can supply such knowledge? Indeed, platonism is a metaphysical thesis that proposes something about the intrinsic nature of mathematical objects—for instance, that they are abstract.

Builes anticipates this response, and argues that *being abstract* is not a ‘genuine’ property. Builes assumes the general distinction between sparse or genuine and abundant properties. On the sparse conception of properties, only a limited number of would-be properties genuinely characterize objects as being thus and so.^9^ Given an abundant conception of properties, on the other hand, things have many properties. The property *being red* you might think, is a genuine property; *being non-red* and *being such that there are no unicorns*, you might think, are not. The latter is completely eliminable from our description of reality, since attributing it to an object tells us nothing about it, nor does it account for any objective similarities in nature. The property *being non-red* is more informative, but is too disjunctive to be a genuine property, since it is equivalent to the property *being blue or green or yellow or*... In this way, we can think of genuine properties as ‘carving nature at its joints’.

Why think that *being abstract* is not a genuine property? After all, if there are both abstract and concrete objects, then the abstract/concrete distinction seems to mark an important joint in reality. Builes contends that, since *being abstract* is defined negatively, as being non-causal and non-spatial, it is no more a genuine property than is *being non- red*. This response, however, puts too much stock into how we happen to talk: *being non-red* is not a genuine property because it is disjunctive, not because ‘non’ is used in its natural-language expression. In contrast, *being non-causal and non-spatial* does not seem disjunctive in the same way. Even though *being non-causal and non-spatial* is expressed negatively, it may yet be a genuine property, in which case, metaphysics will have delivered knowledge of the intrinsic properties of mathematical objects.

Nevertheless, our primary concern here is to develop the view that mathematical objects not only *have* intrinsic properties, but are also *individuated* in terms of them. Properties like *being abstract*, while arguably intrinsic, do not individuate mathematical objects. All mathematical objects are abstract, so the property fails to account for facts concerning their identity and distinctness. It also fails to distinguish mathematical objects from other types of abstract objects, if there are any. What makes mathematical objects different from other abstracta, and what makes one mathematical object different from another, must have something to do with their distinctive mathematical properties. So, while mathematical objects may have intrinsic properties that are discoverable by disciplines other than mathematics, it is not yet clear that any properties discovered in this way will be suitable for individuating mathematical objects.

Here, the platonist can claim that the extrinsic properties of mathematical objects are determined by, or grounded in, some of their intrinsic properties. This picture will be motivated by a general metaphysical thesis according to which all relations must be—at least partly—grounded in the intrinsic properties of their relata. While not everyone will agree with such a principle, those who do will consider metaphysics to have delivered knowledge of the existence of intrinsic properties of mathematical objects. Since these properties are what ground the distinctive mathematical properties of mathematical objects, they are good candidates for the individuation of mathematical objects.

This picture is a form of *Kantian humility* with respect to mathematical objects. The thought is that, while mathematical objects have intrinsic properties, our knowledge of such properties is only indirect, on the basis of the extrinsic properties they ground. We can know that 6 is composite by ascertaining its relationship to 2 and 3; but this relationship is grounded in the intrinsic properties of 6, 2, and 3; properties about which we know nothing other than the fact that they ground that 6 is the product of 2 and 3. The platonist might adopt a global Kantian humility, according to which *all* human knowledge is knowledge of extrinsic properties, or they might adopt instead a local Kantian humility, in which only our knowledge of the *mathematical* realm is like this. Either way, there is a healthy philosophical tradition to appeal to here.^10^

One potential problem with this proposal is that it does not altogether avoid the charge of mysterianism levelled by Builes (2022, pp. 73–4). It embraces the fact that we do not have a positive conception of what the intrinsic properties of mathematical objects are like. However, it does not say that we know *nothing* about these properties. We know that they exist and ground the extrinsic properties of mathematical objects. One might go further and say that this is all we can ever know about the intrinsic properties of any kind of object. For instance, we assign intrinsic properties to physical objects to explain their causal behaviour, and we do not have a positive conception of these properties over and above their role in explaining that behaviour. What is an object’s mass but the intrinsic property that grounds its mass behaviour? Such considerations push us towards a global Kantian humility, and suggest that, if there is a problem here, it is an epistemological one that is not just a problem for platonism.

Perhaps one could argue that there is a relevant difference between our epistemic access to the intrinsic properties of physical objects and mathematical objects, such that we achieve a kind of knowledge of the former that is unachievable in the case of the latter. We won’t take a stand on this. However, this would be an *epistemological* difference between mathematics and the physical sciences. Kantian humility is driven by the metaphysical principle that all relations must be grounded—at least partly—in the intrinsic properties of their relata. There is no reason to think that the envisioned epistemological difference would reflect some relevant metaphysical difference, such that distinctive mathematical properties lack intrinsic grounds that serve to individuate mathematical objects. So, even if our knowledge of the intrinsic properties of mathematical objects is relatively thin, when compared with the knowledge achieved by other disciplines, the view that mathematical objects are intrinsically individuated does not raise any distinctive *metaphysical* problems.

One might object that mathematical relations are *external*, meaning that they are not grounded in the intrinsic properties of their relata.^11^ To illustrate, consider *being the successor of 0* as a property of the natural number 1. The structure of the natural numbers could be instantiated by non-mathematical systems of objects, so long as the axioms of arithmetic are satisfied when suitably interpreted in terms of the relations of the relevant systems. Suppose we line up an infinite collection of rubber ducks, with each subsequent duck placed just to the right of the preceding duck. We can then assign the first duck to ‘0’, and the function *the duck just to the right of x* to ‘*s*(*x*)’, obtaining a model of the axioms of arithmetic, where the duck to the right of the first one satisfies ‘*x* is the successor of 0’. But now suppose we rearrange the ducks, so that the ducks assigned to ‘1’ and ‘2’ are switched. In this situation, one duck loses the property *being the successor of 0,* and another one gains it. But the ducks have not changed their intrinsic properties. So, *being the successor of 0* is not an internal relation; it is external. The same goes for all mathematical properties.

In response, one can distinguish between a genuine mathematical property and the property of merely playing the corresponding mathematical role. A rubber duck can play the role of the successor of 0, relative to a certain isomorphism function φ between the rubber ducks and the natural numbers. The rubber duck is not the successor of 0; it does satisfy the predicate ‘*x* is the successor of 0’ under the interpretation given by φ, but this should be taken not as standing for *being the successor of 0*, but rather for *being the image of the successor of 0 under* φ. The latter is clearly an external relation, so it is no surprise that objects can gain and lose it without undergoing any intrinsic change.

In light of this distinction, we have little reason to think that genuine mathematical properties such as *being the successor of 0* are external. According to the platonist, it is not a matter of arbitrary choice as to which mathematical object has this property. And one available explanation as to why 1, rather than some other number, has this property, is that the intrinsic properties of 1 and 0 make it so. Thus, the platonist who accepts some form of Kantian humility is in a position to claim that certain genuine mathematical properties are grounded in the intrinsic properties of mathematical objects, even if our knowledge of the latter is limited.

To sum up, we have seen that, while there is good reason to think that mathematical properties are extrinsic, this provides no compelling reason to deny that mathematical objects have intrinsic properties. Further, we have seen that there is a defensible view, according to which mathematical objects are individuated by their intrinsic proper- ties. The only sense in which mathematical objects come out as mysterious on this view is that they are individuated by properties that lie, to some extent, beyond our understanding. As we have seen, however, it is not clear to what extent the intrinsic properties of things are within the reach of our understanding. And even if we do know more about the intrinsic properties of physical objects, *metaphysically speaking*, there does not seem to be any distinctive problem for mathematical platonism here.

## Relational individuation

We now consider the view that mathematical objects are individuated by their relations. Structuralism about mathematical objects is an example of this view. It is often characterized as the claim that mathematical objects lack an ‘intrinsic nature’, or that they are ‘merely positions’ in structures. The following paradigmatic expression of the view has been reproduced approvingly by many leading structuralists:

In mathematics, I claim, we do not have objects with an ‘internal’ composition arranged in structures, we have only structures. The objects of mathematics... are structureless points or positions in structures. As positions in structures, they have no identity or features outside a structure. (Resnik, 1981, p. 530)^12^

The key claim is that there is no more to mathematical objects than their structural properties. There is, for instance, no more to the natural number 3 than being the successor of 2, the predecessor of 4, and so on. Mathematical objects are thus individuated in terms of the basic relations of the structure to which they belong. The identity of mathematical objects *depends* on the structure to which they belong.^13^

We respond to three objections against this structuralist conception of the individuation of mathematical objects. The first is that the view results in a vicious circle. The second concerns how mathematical objects are distinguished from other kinds of objects. And the third stems from the general metaphysics of objecthood.

The first objection is that taking the identity of mathematical objects to be dependent on their structure launches a vicious circle. A mathematical structure is a ‘collection’ of ‘objects’ or ‘positions’ that stand in purely structural relations to one another. It is natural to think that which relations are instantiated in a collection of objects depends on which objects comprise that collection. If that is the case, then the identity of a mathematical structure will depend on the identity of its objects. However, according to structuralism, the identity of the objects of a mathematical structure depends on the identity of the structure to which they belong. Given the transitivity of the dependence relation, it follows that structures depend on themselves.^14^

The above argument rests illegitimately on the general assumption that the identity of relations depends on their relata (Linnebo, 2008, p. 71). This might be true in many cases involving ordinary objects: ‘the identity of the relation of *being cousins* seems to presuppose that the relata, human beings, have already and independently had their identities grounded’ (ibid.). However, this defence begs the question against the mathematical structuralist in whose view mathematical objects, unlike ordinary objects, are dependent on their host structures (ibid.). One might worry that our present defense of structuralism and the defense of Kantian humility provided in §2 are in tension. In our defense of Kantian humility, we asserted that an epistemological difference between mathematics and other disciplines provides no reason to think that general metaphysical principles do not apply equally. Yet, above, we accused our objector of begging the question by assuming that mathematical relations are metaphysically akin to relations between ordinary physical objects. If it is generally true that relations between ordinary physical objects are individuated by their relata, and if the structuralist’s distinction between mathematical objects and ordinary physical objects rests merely on an epistemological difference concerning how much we can know about them, then the charge of question begging can be seen as a case of special pleading.

However, this mischaracterizes the dialectical context we are in. The structuralist proposal does not aim to suggest that certain kinds of objects are to be treated differently on epistemological grounds. Rather, the proposal is that we broaden our notion of object to include a different kind of entity that, while playing the semantic role of an object (i.e. serving as the referent of singular terms or as the value of first-order variables), can in many other ways be metaphysically distinguished.^15^ Nor is the envisioned entity one that is wholly unfamiliar. The office of President of the USA is a place in the broader structure of the US Government. It can be thought of as an object, referred to with singular terms, and arguably exists even when no person holds it. Yet, it behaves very differently from any would-be holder of the office, since its existence and identity, and the relations it bears to other offices, depend on the prior existence of the whole structure.^16^ Thus, the structuralist presses entities we already have reason to believe exist into service, showing that they can play the role of mathematical objects adequately, and arguing that this provides an illuminating account of the subject- matter of mathematics. To respond to this by pointing out that mathematical objects and relations behave differently to ordinary physical objects and relations is to miss the point, and indeed to beg the question.

In addition, the structuralist is not forced to accept the thesis that the identity of a structure is dependent on the identity of its objects/positions. They might instead take the identity of a mathematical structure to be dependent in some other way. For example, they could say that, for two systems *A* and *B* of a mathematical theory, the structure of *A* is identical to the structure of *B* if and only if *A* and *B* are isomorphic. Here, the identity of a structure does not depend on the identity of its objects; it depends on isomorphism between the systems instantiating the structure. No circularity arises.^17^

Furthermore, even if one accepts that the identity of a mathematical structure de- pends on the identity of its positions, the circle of dependence is not necessarily objectionable. Consider the natural number 1. On structuralism, the identity and nature of 1 is exhaustively explained in terms of the property *being the second position in the natural-number structure*. If the identity of the natural-number structure were, in turn, dependent solely on the identity of 1, we may have a problem. But it isn’t. If the natural-number structure is dependent on its positions, it is dependent on all of them taken together. While this means that the natural-number structure and its positions are, in a sense, interdependent, there is not obviously anything objectionable about this.^18^

The second objection to relational individuation derives from an oft-quoted passage from Russell:

It is impossible that the ordinals should be, as Dedekind suggests, nothing but the terms of such relations as constitute progressions. If they are to be anything at all, they must be intrinsically something; they must differ from other entities as points from instants, or colours from sounds. What Dedekind intended to indicate was probably a definition by means of the principle of abstraction... But a definition so made always indicates some class of entities having... a genuine nature of their own. (Russell, 1903, p. 86)

Russell wants something to distinguish the natural numbers from other kinds of things. We are owed some explanation as to what makes a number a number, and that requires attributing a nature to numbers that goes beyond their occupying a position in the structure of natural numbers. If we take the wording of this passage at face-value, Russell seems to be suggesting that this will involve attributing intrinsic genuine properties to mathematical objects.

We think that the wording here should not be taken seriously. By saying that numbers must be ‘intrinsically something’, we do not think Russell is suggesting that numbers must have genuine intrinsic properties. Rather, he is merely suggesting that numbers are in possession of something that distinguishes them from other things. Indeed, on Russell’s own view, the required something is not an intrinsic genuine property, but a relation that numbers bear to things ‘outside’ the structure of natural numbers. For Russell, natural numbers are primarily *cardinal*, which is a matter of there existing a one-to-one correspondence that *relates* collections of things-to-be-counted to the cardinal collection. The same goes for Frege’s and the neo-Fregeans’ conception of numbers. According to what is known as Frege’s Constraint, we must define numbers in terms of a principle that explains what kind of entities they *apply* to, and under what conditions such entities determine the same or different numbers.^19^

Hence, even on the Frege-Russell view, the identity of numbers does not depend on their intrinsic properties, if indeed they have any. Rather, numbers are individuated in terms of their applications, and thus, in virtue of the relations they bear to other objects. The difference between structuralism and the Frege-Russell view does not lie in the absence or presence of intrinsic genuine properties; it is down to what kinds of relations determine the identity and nature of numbers: on structuralism, it is the structural relations in which the positions of a structure stand; on the Frege-Russell view, it is the relations involving the objects that can be counted. The significance of this for our discussion is that, even if one agrees with Russell that structural relations are not sufficient to individuate mathematical objects, that does not count against the more general view that mathematical objects are individuated relationally.

The third and final objection we consider stems from the general metaphysics of objects. The claim that there is no more to mathematical objects than their structural properties smacks of the *bundle theory*, according to which objects (particulars or individuals) are merely bundles of their properties.^20^ We can, of course, understand the bundle theory more neutrally: the fundamental facts about what we ordinarily or intuitively qualify as objects involve only properties, making no reference to objects themselves. This leaves open whether objects must be eliminated from ontology, or whether they must be constructed out of their properties.

The objections to the bundle theory are familiar. But one problem which is particularly relevant to our discussion is to explain how *relations* between two or more objects can fit into the relevant bundles.^21^ A bundle of properties has its properties as constituents, in some sense: the redness, roundness, crispness, etc., of an apple are parts of it. But a relation is not part of any of its relata; it is spread across all of them, so to speak. Given that structural relations are indispensable to the individuation of mathematical objects as positions in structures, this objection seems to be particularly pressing for mathematical structuralism.

One way of addressing this concern is to defend ‘eliminativism’ about relations, arguing that all genuine relations are reducible to the intrinsic properties of their relata. This raises a problem for structuralism. Insofar as it entails that mathematical objects are bundles of structural relations, structuralism inherits the problem of accounting for how relations fit into bundles. Worse still, the eliminativist solution is off the table, since structuralism entails that mathematical objects are irreducibly relational.

There are three responses to make here. First, it is not obviously a mark against structuralism that it rules out eliminativism about relations as a potential response to the problem raised above. Eliminativism about relations is far from uncontroversial. It is both contested that all genuine relations are internal—take spatiotemporal relations, for example—and that all internal relations are reducible the intrinsic properties of their relata. Thus, dealing with relations is a problem for everyone who adopts the bundle theory, and eliminativism about relations may be off the table anyway. This diminishes the charge that structuralism renders mathematical objects particularly objectionable.

The second response is to note that the problem of accounting for how *relations* can bundle is a special case of the problem of accounting for how *properties* bundle—another general problem for the bundle theory. The objection is motivated by saying that the properties of an object are, in some sense, part of the object; but the parthood relation is not the folk notion.^22^ So, in what sense, if at all, do properties bundle? Once this is recognized, the sense that there is something *distinctively* problematic about relations diminishes.^23^

The final response is to argue that, once structuralism is properly understood, the problem does not arise. This is achieved by leaning on the metaphysical hierarchy implied by some articulations of structuralism. Recall the much-cited claim that ‘in mathematics... we do not have objects with an ‘internal’ composition arranged in structures, we have only structures’ (Resnik, 1981, p. 530). It is tempting to read this as follows: on the fundamental level, there are no individual mathematical objects at all; there are only entire mathematical structures. These mathematical structures are structured universals, understood as bundles of their constituent mathematical relations. The problem of how relations bundle does not arise here, since the relations characteristic of a given mathematical structure are ‘within’ that structure alone—however ‘within’ is to be understood.

On the present view, individual mathematical objects are derivative entities that are not bundles of relations. As discussed above, they are not really ‘objects’ in our colloquial use of the term; nor do they have mathematical properties in the colloquial sense of ‘have’ reserved for ordinary objects. They are mere positions in the structure, just as structuralists tend to say. The natural number 0, for example, is just the position at the ‘near end’ of the structure that is the bundle of the number-theoretic relations. It is the position that something occupies when it is the first element in a ‘simply infinite system’ of objects. In this way, individual mathematical objects emerge, so to speak, in the negative space left by the bundles of the relations that comprise the entire mathematical structure, just as holes emerge in the negative space left by the weaving of fibres into nets. So, in line with the bundle theory, what exist fundamentally are bundles of relations. These relations are not spread across multiple structures, since the relations of each structure are constituents of that structure alone. Derivatively, there are individual mathematical objects. But these are granted the status of ‘object’ by a broadening of the term, and thus are not themselves bundles of relations, so there is no need to contend with the problem of how to fit relations into them. Not only does this avoid the problem raised above, it dovetails with the elaboration of structuralism as the thesis that the identity of mathematical objects depends on the structure to which they belong.^24^

We have shown that taking mathematical objects to be relationally individuated does not raise any distinctive metaphysical difficulties. The kinds of problems that arise for structuralism are the same kinds of problems that arise for the metaphysics of objects in general. Thus, this kind of view does little to substantiate the claim that mathematical objects are particularly mysterious.

## Substantial individuation

The final view we will consider is that mathematical objects are individuated independently of their intrinsic, relational and structural properties. This marks a decisive break from the other views considered in this paper. According to the view discussed in §2, mathematical objects may be individuated by intrinsic properties that ground or deter- mine their structural properties. According to the view discussed in §3, mathematical objects are individuated by their structural relations. The final view to consider is that mathematical objects are individuated, not in terms of their properties or relations, but in terms of their underlying substance. The identity and distinctness facts concerning mathematical objects are grounded, but not in terms of the qualitative makeup of those objects.

First, we should point out that substantial individuation is, as its title suggests, a way of *individuation*, and so it is different from the position of Leitgeb and Ladyman (2008) and Shapiro (2008) according to which facts concerning the numerical identity and distinctness of mathematical objects are fundamental, and hence, are not to be grounded in some further facts. In their view, identity and distinctness do not require any metaphysical explanation in terms of more fundamental facts. However, as we have mentioned in §1, our working assumption is that identity facts are not fundamental, and thus require metaphysical explanation. According to substantial individuation, on the other hand, there is indeed something that grounds the identity and distinctness facts, even if this task is not undertaken by the properties and relations of the relevant objects.

We will elaborate this position in terms of *bare particularism*, according to which every object, in addition to its properties, has as its constituent a bare particular or ‘substratum’. Underlying the façade of the properties of an object, there is some further entity that is responsible for the possession and the instantiation of the properties of the object. There is thus a sharp contrast between bundle theory and bare particularism. According to the former, objects are constituted entirely by their properties, in the sense that what we normally describe as individual objects are just their qualitative properties that are somehow bundled together. According to bare particularism, however, in addition to their properties, objects have an additional constituent—i.e. their bare particular—which belongs to a distinct ontological category. Thus, the role of bare particulars in this picture is to both *individuate* and to *unify*. They individuate, in the sense that they supply facts concerning the identity and distinctness of the relevant objects: there is a non-property constituent of each object in virtue of which it is the object it is, and is numerically distinct from those objects that do not have that constituent. They unify, in the sense that they are the real bearers of the properties associated with objects—they are the pin cushions in which the properties (the pins!) are stuck.^25^

Bare particulars are called ‘bare’ particulars for a reason: they are sometimes said to have no properties whatsoever. But this requires qualification, since everything has properties. Something without properties would have the property *having no properties*; so the very idea of a propertyless thing is contradictory. There are at least two standard responses to this puzzle in the literature. The first, due to Sider (2006), is to draw on the sparse view of properties, and suggest that, while bare particulars have abundant, non- genuine, properties, they lack sparse, genuine properties. *Having no genuine properties* is not a genuine property, so no contradiction arises. Another response is to deny that bare particulars lack properties. Indeed, one of the theoretical roles mentioned above, namely unifying, seems to require that they have properties, at least in some sense of ‘have’. Following Wildman (2015), we can say that objects *possess* properties, while bare particulars *bear* properties; and, taking *bear* to be primitive, we can define *possession* in terms of *bearing* as follows: an object possesses the property *F* if and only if it has a proper constituent that bears *F*. On this view, bare particulars are bare only in the sense that they do not possess properties. So, the position we are considering here is that the identity of a given mathematical object is grounded in the identity of its non-qualitative constituent that bears its properties. Further, the fact that one mathematical object is distinct from another is grounded in the fact that their non-qualitative constituents are distinct.

Some philosophers have found bare particulars puzzling. For example, Lowe (2003) complains that bare particulars are qualitatively indiscernible, and thus ‘self- individuating’, which he finds mysterious. But even if one shares Lowe’s sense of mystery, there is nothing obviously *incoherent* about the notion of entities whose identity and distinctness are brute. One might suggest that the sense of puzzlement some feel towards the idea of self-individuation warrants a default skepticism towards the possible existence of bare particulars. But it is hard to maintain such skepticism given that both mathematical and scientific theories seem committed to *utterly indiscernible* entities; that is, entities which possess all the same properties as each other, and stand in the same relations to all other entities, and yet are numerically distinct. Take, for example, an edgeless graph with just two distinct nodes. Since the nodes of this graph are numerically distinct and yet utterly indiscernible, they violate even the weakest forms of the Principle of Identity of Indiscernibles. The practice and opinions of competent scientists and mathematicians provides at least some *prima facie* support for the view that the existence of self-individuated entities is possible. It thus seems that not only is the concept of self-individuation—and hence, brute individuation—coherent, we have some reason to think it is instantiated.

However, Builes points out that the impossibility of bare particulars can be derived from certain metaphysical views. He offers the example of the bundle theory, and writes:

Various different versions of the bundle theory have been defended... All of them, however, imply that bare particulars are *impossible*. If there are no properties to bundle together, then there is no corresponding object. (Builes, 2022, pp. 68–9; emphasis added)

We accept that the truth of the bundle theory would rule out the actual existence of bare particulars, but whether or not it would rule out the *possibility* of bare particulars crucially depends on the question of whether fundamental metaphysical theses are necessary or contingent, which is a matter of debate.^26^ Moreover, the bundle theory provides us with reasons to rule out the existence of bare particulars only to the extent that we have reason to believe the bundle theory in the first place. To show that we should, on balance, prefer the bundle theory is a very ambitious project; to show that the bundle theory is necessarily true is very much more ambitious. But this is what would be required to appeal to the bundle theory to establish the impossibility of bare particulars.^27^

All of this suggests that it goes too far to claim that bare particulars *cannot* exist. But are they nevertheless mysterious? And, more to the point, does the idea that mathematical objects are—or at least contain—bare particulars pose any distinctive problems? We suggest an affirmative answer to this question.

According to the substantial individuation view, mathematical objects are individuated independently of their intrinsic properties and relations. The view thus severs the link between the identity and distinctness facts concerning a mathematical object and the object’s distinctive mathematical properties. This seems odd, to say the least. One might think that what accounts for the numerical identity and distinctness of 1 should have something to do with, for example, its being the successor of 0, or with some facts about its applications in counting singly-instantiated concepts. More importantly, this view renders the relationship between mathematical objects and their distinctive mathematical properties mysterious. This can be shown by the following dilemma. Either mathematical objects have their mathematical properties necessarily, or they do not. If they do, then the present view lacks the resources to explain why they do. After all, the identity of the number 1 is, on the present proposal, is a brute fact that has nothing to do with the properties it has. So, we are forced to posit a further brute matter of fact: that, as a matter of necessity, the number 1 has the property *being the successor of 0*. And the same goes for all mathematical properties. The resulting picture is one according to which mathematical objects have an intimate connection to certain properties, but there is no explanation as to why.

One could avoid this horn by claiming that mathematical objects have their properties—mathematical and non-mathematical—contingently. This runs against the platonist orthodoxy; but, more importantly, it does not help. On the present view, there are actual matters of fact concerning which mathematical objects have which mathematical properties. *Ex hypothesi*, these actual matters of fact could have been otherwise. Yet there is nothing that could explain why the facts turned out this way, rather than some other way: why does the number 1 have *being the successor of 0*, rather than *being the successor of 7*? There is no sensible answer that can be given on the present view. Thus, the view that mathematical objects are substantially individuated does raise distinctive metaphysical difficulties. If mathematical platonism is committed to the view that mathematical objects are, or are akin to, bare particulars, the objection that mathematical objects are mysterious has some substance.

However, we shall see in the following section that there is little reason to think that mathematical platonism is so committed.

## The alleged necessity of nominalism

Builes (2022) aims to explain why mathematical nominalism is metaphysically necessary, and thereby sharpen “the widespread intuition that mathematical objects are somehow ‘spooky’ or ‘mysterious’” (2022, p. 74). Builes’ argument runs as follows:

1. Necessarily, there are no bare particulars.
2. Necessarily, if there are abstract mathematical objects, then there are bare particulars.
3. Therefore, necessarily, there are no abstract mathematical objects.

We argued in §4 that we have little reason to accept (1). However, we also argued that taking mathematical objects to involve bare particulars does raise distinctive meta- physical problems. Thus, the argument may, with suitable adjustment, still succeed in sharpening the nominalist objection that mathematical objects are mysterious, provided that (2) is well-motivated.

Builes’ argument for (2) can be summarized as follows: (i) the only defensible form of platonism is structuralism about mathematical objects; (ii) structuralism about mathematical objects entails that mathematical objects have no genuine intrinsic properties; (iii) therefore, platonism, in its most defensible form, entails the existence of bare particulars.

Crucially, the argument relies on a definition of bare particulars as entities that lack genuine intrinsic properties. Builes’ argument for (i) begins with the observation that ‘mathematics is the study of purely structural or relational features of things’ and ‘does not concern itself with the intrinsic nature of the particular objects that stand in such relations’ (Builes, 2022, p. 70). As discussed in §2, we accept this. Builes then presents a dilemma. Either we take mathematical objects to have intrinsic properties that do not form part of the subject-matter of mathematics, or we adopt structuralism about mathematical objects. He rejects the first horn, and takes structuralism to entail that mathematical objects are bare particulars. However, our preceding discussion shows that this dilemma maps the logical terrain poorly.

Take the first horn. It should be clear from §3 that if one denies that mathematical objects have genuine intrinsic properties, one is not thereby committed to structural- ism about mathematical objects. Indeed, the Frege-Russell conception of the natural numbers as cardinal numbers is compatible with the view that they have no genuine intrinsic properties, and yet they are not individuated by their structural relations. Nevertheless, Builes’ argument only requires that platonism is committed to the view that mathematical objects lack genuine intrinsic properties—and structuralism and the Frege-Russell view are both committed to this view. The more pressing question is whether denying that mathematical objects have genuine intrinsic properties commits one to the existence of bare particulars. The answer is ‘no’. In §3, we argued that mathematical objects can be individuated in terms of their characteristic structural relations, and so, there is no need to posit bare particulars to individuate mathematical objects or unify their properties. *Contra* Builes, mathematical structuralism is not committed to the existence of bare particulars.

At this point, Builes may object that we are employing a different definition of ‘bare particular’ to the one employed in his argument. For Builes, a bare particular just is an entity that has no genuine intrinsic properties. Thus, structuralism’s denial that mathematical objects have genuine intrinsic properties is, by definition, a commitment to bare particulars, on Builes’ definition. However, given our discussion in §4, Builes’ definition can be questioned. Bare particulars are characterized by their theoretical role, which is to individuate and unify. A more accurate definition would be that a bare particular is a self-individuating entity that has no properties as its constituents. As our discussion in §3 makes clear, however, mathematical structuralism is not committed to such entities.

Moreover, even if Builes insists on retaining his definition of bare particulars, the result is that mathematical objects, conceived of as bare particulars in Builes’ sense, do not raise any distinctive metaphysical problems, and so, do not substantiate the claim that mathematical objects are mysterious. If our discussion in §§2–4 is correct, then the only picture of mathematical objects that gives rise to distinctive metaphysical problems is the view that mathematical objects are individuated substantially. But, for all Builes’ argument suggests, mathematical objects may yet be individuated in terms of their relations. The second horn of Builes’ dilemma, therefore, fails to substantiate the nominalist objection.

Nor are we forced onto the second horn in the first place. In §2, we showed that the view that mathematical objects have and are individuated by their intrinsic properties is defensible, and that Builes’ reasons for thinking otherwise are not convincing. The platonist can, therefore, happily grasp either horn of Builes’ dilemma.

## Conclusion

In this paper, we have explored the prospects for substantiating the nominalist motif that mathematical objects are mysterious by exploring how they are individuated. We have examined three views on the matter: that they are intrinsically individuated; that they are relationally individuated; and that they are individuated in terms of their underlying substance. We have found that the nominalist objection gains support only to the extent that the platonist is under pressure to accept the third mode of individuation. We then have identified a recent argument from Builes as potentially providing pressure in that direction, but found that it is unsuccessful. We have concluded that, at least as far as the question of how mathematical objects are individuated, the nominalist objection has no bite. Whether it can be fleshed out in another way is a question for another time.

## Footnotes

1 For more discussion on the epistemological challenge, see Benacerraf (1973), Field (1989, pp. 25–30, 230–9), Clarke-Doane (2016), and Liggins (2018).

2 For more discussion on this notion of individuation, see Lowe (2003) and Linnebo (2008, 2009).

3 Our methodology, following Ladyman (2007) and Caulton and Butterfield (2012), among others, is as follows. Suppose that *a* and *b* are two objects. Then, we have four ways for *discerning a* and *b* relative to a suitably regimented language. The first two modes of discernment, known as ‘monadic discernibility’, contain formulas only with one free variable. The second two modes contain formulas with two free variables. These correspond to what is often called ‘relational discernibility’. And if *a* and *b* are not even relationally discernible, it is said that they are ‘utterly indiscernible’. (See §4 for more discussion on the notion of utter indiscernibility.) In this paper, we have shifted our focus from discernibility relative to a language to metaphysical individuation relative to properties and relations. All the same, we do not claim that the modes of individuation to be explored below are exhaustive.

4 See Wilson (2014) for discussion.

5 See Litland (2022) for a defence.

6 This view has been defended by Wiggins (2001).

7 For a helpful discussion of the extrinsic-intrinsic distinction and related references, see Marshall and Weatherson (2018).

8 See Korbmacher and Schiemer (2018) for various characterizations of the notion of structural property in the context of mathematical structuralism. In what follows, nothing substantial depends on the exact characterization of this notion.

9 The terminology of abundant and sparse or genuine properties originates in Lewis (1986, pp. 59–69). The general idea is due to Armstrong (1979).

10 For more on Kantian humility, see Langton (2001). Russell (1919, pp. 61–2) advocates a form of Kantian humility about mathematics.

11 Here, we draw on Armstrong’s (1978, pp. 84–5) distinction between internal and external relations. See also Lewis (1986, p. 62).

12 For similar views, see Shapiro (1997, pp. 73–5), Parsons (2004, p. 57), Linnebo (2008), and Leitgeb (2021).

13 See Linnebo (2008) for a development and a partial defence of this conception of ontological dependence. See also Shapiro (2008, §4) and Sider (2020, ch. 3).

14 This objection has originally been put forward by Hellman (2001, pp. 193–4). See Linnebo (2008) for more discussion.

15 See Reck and Price (2000, §7) for discussion of this point.

16 See Shapiro (1997, p. 9) for the analogy with social organizations.

17 Following Shapiro (1997), systems are ordered (*n*+1)-tuples consisting of a domain of objects—identifiable independently of the system to which they belong—and relations and functions on this domain. Structures, however, are what isomorphic systems ‘have in common’. For more discussion on this conception of the individuation of mathematical structures, see Shapiro (1997, p. 93), Linnebo and Pettigrew (2014), and Leitgeb (2021).

18 See Thompson (2016) for a defence of metaphysical interdependence, with some compelling examples.

19 For more recent discussion on Frege’s Constraint, see Hale (2016) and Snyder, Samuels, and Shapiro (2018).

20 This accords with MacBride’s (2006, p. 65) construal of Shapiro’s structuralism.

21 For more on this objection, see Hawthorne and Sider (2002, §1) and Sider (2020, §3.9).

22 Paul (2006, 2012) argues that the relationship between ‘objects’ and their properties should be understood in terms of mereological composition. See Keinanen and Tahko (2019) for a response.

23 Some articulate the relevant notion of parthood in terms of *co-location*. But this approach would not be suitable for a bundle-theoretic account of *mathematical* objects, anyway, since mathematical objects lack location. Thankfully, there are other accounts that do not invoke location, and have been deployed in making sense of irreducible relations. We submit that some such accounts, if they work, may offer a way of spelling out what mathematical objects are on the structuralist proposal.

24 In fact, in Shapiro’s view, positions of mathematical structures are ‘simple’ entities: they are ‘atoms’ with no proper parts, so they are not bundles. See Shapiro (2008, pp. 302–3; 305) See Assadian (2024) for more discussion on mathematical structuralism and bundle theory.

25 For more discussion on bare particulars, see, for instance, Sider (2006, 2020, §3.10) and Wildman (2015).

26 Builes (2022, p. 68) also offers a brief conceivability argument against bare particulars. A proper discussion of this issue goes beyond the scope of this paper.

27 See Miller (2020) for more discussion. It should also be noted that the bundle theory is not the only motivation against bare particulars. As Builes (2022) points out, there are other views—for example, Dasgupta (2009)—that also imply the following thesis: necessarily, if that view obtains, then there are no bare particulars. All the same, our point about the bundle theory, *mutatis mutandis*, applies to these views, too.
